# Based on a multi-agent system for multi-scale simulation and application of household's LUCC: a case study for Mengcha village, Mizhi county, Shaanxi province

**DOI:** 10.1186/2193-1801-2-S1-S12

**Published:** 2013-12-11

**Authors:** Hai Chen, Xiaoying Liang, Rui Li

**Affiliations:** Institute of Soil and Water Conservation under Academia Sinica and Ministry of Water Resources, Northwest Sci-Tech University of Agriculture & Forestry, Yangling, Shaanxi China; Department of Urban and Resource Sciences, Northwest University, Xi’an, China

**Keywords:** MAS, household, multi-scale, the framework of the decision-making

## Abstract

Multi-Agent Systems (MAS) offer a conceptual approach to include multi-actor decision making into models of land use change. Through the simulation based on the MAS, this paper tries to show the application of MAS in the micro scale LUCC, and reveal the transformation mechanism of difference scale. This paper starts with a description of the context of MAS research. Then, it adopts the Nested Spatial Choice (NSC) method to construct the multi-scale LUCC decision-making model. And a case study for Mengcha village, Mizhi County, Shaanxi Province is reported. Finally, the potentials and drawbacks of the following approach is discussed and concluded. From our design and implementation of the MAS in multi-scale model, a number of observations and conclusions can be drawn on the implementation and future research directions. (1) The use of the LUCC decision-making and multi-scale transformation framework provides, according to us, a more realistic modeling of multi-scale decision making process. (2) By using continuous function, rather than discrete function, to construct the decision-making of the households is more realistic to reflect the effect. (3) In this paper, attempts have been made to give a quantitative analysis to research the household interaction. And it provides the premise and foundation for researching the communication and learning among the households. (4) The scale transformation architecture constructed in this paper helps to accumulate theory and experience for the interaction research between the micro land use decision-making and the macro land use landscape pattern. Our future research work will focus on: (1) how to rational use risk aversion principle, and put the rule on rotation between household parcels into model. (2) Exploring the methods aiming at researching the household decision-making over a long period, it allows us to find the bridge between the long-term LUCC data and the short-term household decision-making. (3) Researching the quantitative method and model, especially the scenario analysis model which may reflect the interaction among different household types.

## Introduction

### Scale and multi-agent system model of land use change

Questions of land use/land cover change (LUCC) have attracted interest among a wide variety of researchers. Among these, geographers and natural scientists have taken the lead in developing the land use/land cover scenario simulation model [1]. At present, some researchers have proposed series of valuable scenario simulation models, such as Conversion of Land Use and Its Effects model, Cellular Automata model, System Dynamic model and City Expanding Model [2–4]. However, less attention has been paid in the main subject decision-making of land use/land cover change and their behavior. Multi-Agent Systems (MAS) offer a conceptual approach to include multi-actor decision-making into models of land use change, it is particularly well suited for representing complex spatial interactions under heterogeneous conditions and for modeling decentralized, autonomous decision-making [5].

MAS model is applied to model the land use/land cover change at various spatial scales at present. Arend Ligtenberg and Liu Xiaoping, for example, used MAS model to simulate LUCC of urban [6, 7], Diego Valbuena and Jean-Christophe Castella carried out research on regional LUCC [8, 9]. Some research mainly concentrated on micro scale. Bockstael and Tom P. Evans have explored MAS to simulate land use change of the household [1, 10]. Irwin gives a duration model to understand the economic process that underlies land use change -- namely, the human behavioral component [11]. Although the MAS model provides a flexible platform to experiment at various spatial scales, it still needs further research on communication of households, the interaction among households, environment and market, the learning and adopting behavior of households at micro scale [5, 12].

Scale issues have significant implication for the design and application of models of LUCC [13, 14]. At present, there are 3 methods in multi-scale topic research. Based on the mechanism analysis, the scale transformation rule is explored from the individual decision-making to group decision-making in the first method [15]. Through changed the input data resolution, the scale issue is discussed in the second method [16]. The third method combines other model, for example, CA or CLUE, with MAS to analysis multi-scale issue [2, 16]. Through the transformation mechanism between different scales being clear in the first method, it is difficult to explicit spatially the decision-making. The second method may explicit spatially the decision-making, but the explanation power between LUCC and decision-making is not strong. The third method may reveal the relationship between the complexities and the decision-making, which is simple to deal with the agent interaction.

### The study area

Mengcha village locates at Mizhi County, Shaanxi Province, which is in north of China. The range is 110° 6′00″-110 ° 9′30″E longitudes and 37°45′50″-37°47′30″N latitude. Mengcha village locates at Temperate Semi-arid Region, annual average temperature is 8.5°C, frost-free period is 162 days, and mean annual precipitation is 415.6 mm. There are 150 households and the population of the village is 820. The land area is approximately 36 hectare, consisting of 3 land use types: hilly area land, valley land and dam land. The economic forest - jujube tree is planted in the hilly area land, corn is mainly planted in the dam land, and cash crop is planted in the valley land.

The project of turning the cultivated land into forests or grasslands began in 2000 at Mengcha village, the land use pattern changed greatly. In 2003, all the hilly area land was returned; the growing of cash crops in the valley land was influenced by the market. Compared to the hilly area land and valley land, the crops planted in the dam land are relatively stable, mainly corn, occasional planting glutinous broom corn and black soya bean. The household type has changed from a single type --- the planted household to 5 types (the contractor household, the planted household, the worked household, the part-time household and the level government). The multi-function of multiple land use types, various kinds of household types and the project of turning the cultivated land into forests or grasslands as well as the market effect provide a better platform to research the LUCC decision-making of household in Mengcha village. The intelligent agents have the following characteristics:

1. The contracted household mainly plants economic forest. Because of the contracted household making contract with other households, the interaction includes not only among the contracted households, but with other household types.
2. The planted household mainly plants valley land. The interaction of the planted household involves the interaction among the planted households and the interaction with other household types.
3. The worked household mainly works for a living. This household type gives their valley land and hilly area land to the planted household and the contracted household respectively.
4. The part-time household mainly works for a living through seasonal work and planted valley land. They give their hilly area land to the contracted household.
5. In comparison with other household types, the grass-roots level government is a special type. Local government officials can contact with individual households and the whole households, and predominates in policy-making of contracting the economic forest and renting the valley land.

As the main body of the land use, households' decision-making determines the change of the micro land use change. The household decision-making type is closely related to land use change. Therefore, it finds a feasible way to reveal the mechanism of the scale transformation that analyzing from the household decision-making type. For the sake of simplicity and typicality, this paper chose the planted household which is less affected by other household types and which is easily available in amount and variety in our research.

## Multi-scale LUCC decision-making model

### The transformation mechanism of multi-scale decision-making

A broad array of data sources are used to represent key dynamics in the land use decision-making. These data sources include economic/price, land use, and agricultural census information. Crop price, melon and fruit price, and vegetable price are considered exogenous and uniform for all households. Crop price for corn is an average derived from Mizhi county Agricultural Census data sources. Our land use data does not allow us to discriminate with a sufficient level of accuracy what melon and fruit are being grown and what vegetable species being grown in agricultural areas. Thus, we simplify the model to a crop class, a single melon and fruit class, and a single vegetable class in our model runs.

Land ownership boundaries were derived from the parcel maps provided by the Mizhi County Land Resource office for 2000. These parcel boundaries provide the essential information. Therefore, agents are assigned to the landscape over time. In order to reveal the land use change, the household interview was conducted in 2007 and 2008.

**Figure 1:**
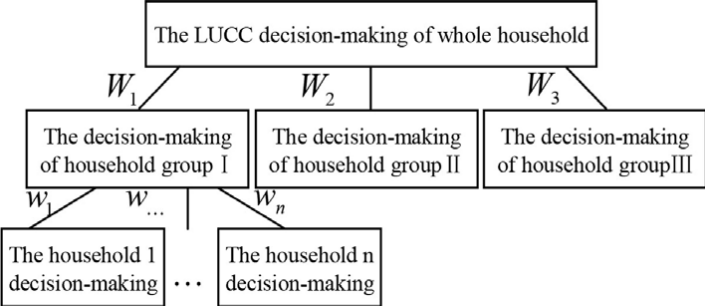
**The scale transformation mechanism from individual household decision-making to the whole household decision-making**.

The rule of the multi-scale transformation is the key to realize the transformation from the individual household to the whole household. In this paper, the NSC is used for the scale transformation. Benenson and Torrens use NSC to research the urban residential choice, their scale transformation methods provide a useful reference [17]. The transformation mechanism is shown in Figure 1.

(1) The decision-making of the whole households is affected by the household groups. The effect weight coefficient *W*_*ikt*_ of each group is described as:

Where *i* is household group *i*; *k* is the land use type *k* ;

(2) The decision-making of the household groups is affected by the households. The effect weight coefficient *w*_*ikt*_ of each group is described as:

Where *j* is household *j* ;

### Model description

#### The whole household decision-making model

The whole household decision-making is related to the household groups' decision-making and their weight. The whole household decision-making formula is:1

Where *wholehousehold*_*kt*+1 _is the decision-making of the *k* land use type of the whole household at time *t*+1; *W*_*ikt*_ is the influence coefficient of the *k* land use type of the household group *i* at time *t*; *householdtype*_*ikt*_ is the decision-making of the *k* land use type of the household group *i*(*i = 1, 2, ..., n*) at time *t*.

The influence coefficient formula is described as following:2

Where *Area*_*ijkt*_ is the land area of the *k* land use type of the individual household *j* in the household group *i* at time *t*; *Income*_*ijkt*_ is the earnings of the k land use type of the individual household *j* in the household type *i* at time *t*.

#### The household groups' decision-making model

The household groups' decision-making is related to the individual household decision-making and their weight. The formula of the household groups' decision-making is:3

Where *w*_*ijkt*_ is the influence coefficient of the *k* land use type of the individual household *j* in household group *i* at time *t*; *household*_*ijkt*+1 _is the decision-making of the *k* land use type of the individual household *j* in the household group *i* at time *t*+1.

The formula of the influence coefficient *w*_*ijkt*_ is described as following:4

#### The individual household decision-making model

Combined with the actual situation in the household investigation, household land use intention model comes with a number of assumptions:

- ■ The short-term land use decision-making is considered.
- ■ There is little difference in planting technique among households.
- ■ The natural conditions of the valley land are similar.
- ■ During the study, planting technology does not change.

Under the framework of the rational decision-making, this paper attempt to construct the individual household decision-making, it is the function of the factor of the institute, market, households themselves and the interaction between households. The individual household decision-making formula is described as following:

Where  is the decision-making of the  land use type of the individual household  at time t+1;  is the influence of the policy to the  land use type of the individual household  in the household group  at time t. In this paper, the policy influence is mainly referred to the turning of cultivated land into forests or grasslands policy and the enactment of rent;  is the effect to the  land use type of the individual household  in the household type  at time t;  is the interaction among the individual household  which has the  land use type in the household group  at time t;  is the influence coefficient of the policy, the market and the interaction among households respectively, and .

The turning of cultivated land into forests or grasslands policy began in 1999. According to the approved area, the government appropriate food subsidies, planting seeds and cash benefit to the household. In study area, this policy is aimed at the hilly area land and sloping land. This paper concerns about the valley land, therefore, its land use pattern has less affected by this policy. Because quite a number of the valley is dominated by the grass-roots level government, the rent is relative stable.

The institutional factors provide the macro background. Because of the stable rent and the effect range of the policy, the effect of the institutional factors to the household decision-making is equal to zero.

The factor of the household themselves mainly includes rotate, risk averse and the crop importance. The formula of decision-making of the individual household is described as following:

Where *rotate* is the rotate factor, this paper mainly refers to the watermelon rotation to corn or vegetable; *rlude-risk* is the factor of risk averse; *crop-importance* is the factor of crop importance;

■ The rotate factor.

In this paper, the household plants three major crops: corn, vegetables and watermelon. Because of consuming larger soil fertility, the watermelon can not be planted in the same filed for two consecutive years. In the study area, the household has the habit of planting watermelon. Therefore, the planting period of the field of corn or vegetable is beyond 2 years, the field is rotated to watermelon. Above can be expressed as following:

  If "the planting crop is watermelon" then

    watermelon → corn

   Or watermelon → vegetable

  If "the planting crop is corn or vegetable" then

   If the planting period ≥ 2

    corn or vegetable → watermelon

   If the planting period < 2

    corn or vegetable → corn or vegetable

■ The factor of the risk averse

Because of the unknown of the future climate, the water source and market, although seeking to maximize benefit, the households make their cultivation as far diversification as possible in order to reduce the planting risk. Therefore, this paper comes with a number of assumptions; which can be described as the following:

  If "the count of the household field" ≤ 2 then

    "the land use can not change"

  If "the count of the household field" > 2 then

    "cultivation as far diversification as possible"

■ The Crop-importance factor

In this paper, the crop-importance factor is referred to the importance contrast between corn and vegetable. The contrast includes the cattle, the household planting habit, the crop benefit and the land use area. Under satisfied the factor of the rotate and the risk averse, the household will planting corn as possible if having cattle, or contrasts the importance between corn and vegetable. The crop importance judgment is described as following:

 If "satisfied the factor of the rotate and the risk averse" then

  If "the household has cattle" then

   If the household planting crop and vegetable simultaneously then

    (corn or vegetable) → corn

 Or

 If "the household only plants watermelon and corn,

 or watermelon and vegetable" then

End if

Where  is the importance of the  land use type of the individual household  in the household type  at time t. its formula is described as following:5

The formula interaction among households is described as following:6

where:

 is the influence coefficient among households.

Its formula is:7

The decision-making of each household is affected by the market. In order to find out a quantitative expression of the influence of the market to the decision making of household, 2 assumptions are made as follows:

The pattern of the valley land reflects the current market information;

The household masters the earning information of all kinds of the land use types.

There is a difference influence of the market for each household type. Through analyzing the LUCC pattern of each household type and their earning from the land, we can obtain the whole effect to each land use type by the market. The formula is:8

We can use formula (1) to obtain the current land use type importance of the planted household; and combining the formula (6), the market effect to each type land use is calculated. The formula is described as follows:9

Therefore, the formula of the finial decision-making of the individual household is described as:10

Where  can use the economic crop land change within two years to express market effect. Namely,11

## Results

### The decision-making of the individual household

According to the formula (5), the land use importance is calculated (see Table 1). Because it involves a number of households, only typical kinds of households are shown in Table 1. We can see from Table 1:

**Table 1:** The crop importance of the planted households

The typical household	Corn	Vegetable	Other crops
1	0.06	0.94	0.00
2	0.13	0.87	0.00
3	0.73	0.27	0.00
4	0.70	0.30	0.00
5	0.00	0.01	0.99
6	0.00	0.05	0.95
7	0.28	0.47	0.25
8	0.42	0.43	0.15
*The whole average*	*0.38*	*0.45*	*0.17*

- ■ From the perspective of crop importance, there are 3 planted types in Meng-cha village: the type of mainly planting the corn, vegetable and other crops.
- ■ From the perspective of whole households, the rank of land use importance of the planted household is vegetable field, corn field and other field.

### The interaction between households

 is calculated (see Table 2) according to the formula (6) and (7). From the tab.2, we can see:

**Table 2:** The interaction between households

	The interaction coefficient ()			The interaction among households ()		
	**Corn**	**Vegetable**	**Other Crops**	**Corn**	**Vegetable**	**Other crops**
1	0.94	0.01	-	0.01	0.05	-
2	0.87	0.09	-	0.11	0.07	-
3	0.37	0.71	-	0.22	0.20	-
4	0.82	0.69	-	0.09	0.23	-
5	0.99	-	0.00	0.01	-	0.00
6	0.95	-	0.04	0.04	-	0.04
7	0.65	0.88	0.75	0.09	0.12	0.19
8	0.86	0.77	0.84	0.19	0.24	0.09

■ The land use importance of all kind of types in the planted household shows a contrary tendency to the influence coefficient. Namely, the more the land use importance of individual household is, the less the influence coefficient is.

■ Compared to ,  of all planted households becomes a little slowdown. It is because the effect of .

■ It might also be noted that the household type is closely related to the intention pattern and degree. Using the  and , the effect of the difference household types are clearly revealed. Compared to household 7 and 8, for example, the household 1 differs obviously in the effect of the interaction between the households. The importance of the vegetable field of household 1 more important than the household 7 and 8.

### The market factor

According to the formula (8) and (9), the market effect to the whole crops planted and the individual household planted is calculated respectively. The result can be shown in Table 3.

**Table 3:** The effect of the market to the whole household and to the individuals

The difference level effect of the market		Corn	Vegetable	other crops
The effect to the whole household	1	0	0	0
	2	0	0	0
	3	-0.25	0.21	0
	4	-0.03	0.19	0
	5	0	0.51	-0.84
	6	0	0.45	-0.79
	7	0	0.34	-0.09
	8	0	0.27	0
The effect to the whole household		0.35	0.49	0.16

From Table 3 we can see:

- ■ From the perspective of the whole crops planted, the effected order is vegetables, corn and other crops.
- ■ If the effect is positive, it indicates this land use type should be expanded, and the bigger the numerical value of the effect is, the bigger the market effect to the land use will be. If the effect is negative, it indicates this land use type should be reduced, and the bigger the absolute value of the effect is, the bigger the market effect to the land use is.
- ■ The household types are closed related to the market effect to land use. Through

and , the market effect to the difference household types shows clearly.

### The final decision-making of the household

The interaction of the households and the weight coefficient of the market are calculated according to the formula (11). The final decision-making of the household is calculated, the result sees Table 4 according to the formula (10).

**Table 4:** The final decision-making of the household

	The final decision-making of the household ()			The standardization of the final decision-making ()		
	**Corn**	**Vegetable**	**Other crops**	**Corn**	**Vegetable**	**Other crops**
1	0.11	0.95	0.02	0.11	0.88	0.01
2	0.24	0.95	0.02	0.20	0.78	0.02
3	0.78	0.69	0.02	0.52	0.46	0.02
4	0.83	0.70	0.02	0.54	0.45	0.01
5	0.02	0.08	0.16	0.08	0.31	0.61
6	0.04	0.54	0.20	0.05	0.69	0.26
7	0.65	0.35	0.47	0.44	0.24	0.32
8	0.43	0.73	0.28	0.30	0.51	0.19

Combined with Table 1, we can see from Table 4:

- ■ The decision-making of other kinds of households is changed because of the effect of the market and the interaction of the households. We can see the decision-making of the individual household is not to achieve maximum economic efficiency, namely the decision-making of rational economic men, but to achieve bounded rationality, that is to say, the aim is not to pursue the optimization, but to pursue the satisfaction.
- ■ The method can quantitatively express the effect of the non-planted crops to the household decision-making too.

Contrasting the final decision-making of the household in 2007 with the actual decision-making in 2008, we can derive the accuracy rate of the whole household and the household groups' (described in Table 5).

**Table 5:** The illustrate of the error count and types of the household

The household type	The household count	The error type and count	The accuracy rate
Type1	40	Risk averse(2), decision-making(1)	92.5%
Type2	30	Risk averse(2), decision-making(1)	90.0%
Type3	13	0	100%
Total	83	6	92.7%

According to the reason of the error, there are 2 error types: the risk aversion violation and household decision-making error. Among these, there are 4 households which their decision-making is correct but violate the risk averse principle. There are 2 households which their decision-making is error. The total accuracy rate is 92.7%.

Where the type 1 is the household of the mainly planting the vegetable; the type II is the household of the mainly planting corn; the type 3 is the household of the mainly planting other crops.

### The decision-making of the household groups and the whole household

The decision-making of the households groups and the whole household are calculated according to the formula (1)-(4). The results are shown in Table 6.

**Table 6:** The decision-making type of the household groups

Household group type	Corn	Vegetable	Other crops
Household group 1	0.26	0.55	0.19
Household group2	0.39	0.41	0.20
Household group 3	0.32	0.42	0.26
The whole household	0.31	0.47	0.22

The order of the crop importance of the 3 households groups is vegetables, corn and other crops. The difference among 3 groups is: the household group 1 is mainly planning the vegetable; the risk averse is manifested in household group 3; the decision-making of the household group 2 between the other two groups.

Compared to using the simple average of the whole households' decision-making, the household groups' decision-making may reflect the difference in the planting households, and may reveal the decision-making diversity of the household.

The decision-making of the whole household reflect the land use decision-making of the next year. The order of the crop importance of the next year is vegetable, corn and other crops.

### The model verification and validation

**Figure 2:**
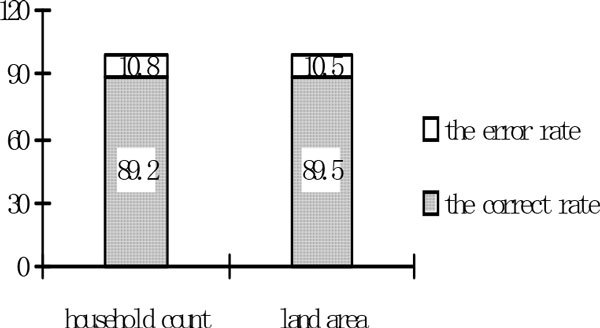
**The error of the decision-making**.

In order to verify the effectiveness of the method based on the NSC, this paper use the data of year 2007 to simulate the land use decision-making of the household of the year 2008. Combined with the actual data of year 2008, the error type and error count of the household decision-making and the spatial distribute of the field is derived (the results as shown in Table 7 and Figure 2).

**Table 7:** The illustration of the errors

Household group type	The household count	The error type and count	The accuracy rate
Household group 1	40	Risk averse (2), decision-making (3)	87.5%
Household group 2	30	Risk averse (2), decision-making (2)	86.7%
Household group 3	13	0	100%
The whole households	83	The above-mention (9) + decision-making (1)	87.9%

- ■ From the perspective of household groups, the error rate is 10.8%, and has two error types: the risk aversion violation and household decision-making error. Among these, there are 4 households which their decision-making is correct but violate the risk averse principle. (2) There are 5 households which their decision-making is error. The total accuracy rate of the household groups is 89.2%.
- ■ From the perspective of the whole households, the accuracy rate is 87.9%. Therefore, the method based on NSC can better reflect the multi-scale farmers in land-use decision-making.
- ■ From the perspective of error reason, the reason of the decision-making error of the household is rotate crops among difference fields. The risk adverse principle is rigid.
- ■ From the perspective of the area of the land, the error rate is 10.5%, the error area of decision-making and risk averse is 8.0% and 2.5% respectively. In this paper, if the decision-making of the household is error, then his own land is calculated in the error area.

## Conclusions

From our design and implementation of the MAS in micro scale model, a number of observations and conclusions can be drawn on the implementation:

1. Using the LUCC decision-making and multi-scale transformation framework provides, according to us, a more realistic modeling of multi-scale decision-making process. In this paper, three level LUCC decision-making model is constructed, the result can be better simulated the actual household decision-making. Therefore, the multi-scale LUCC model in this paper can well reflected the multi-scale land use decision-making.
2. The change of the accuracy rate of the decision-making which from 92.7% in individual household to 87.9% in the whole households, indicates that the NSC method can well reveal the multi-scale transformation mechanism of the land use decision-making.
3. it is more realistic to reflect the actual effect by using continuous function instead of discrete function to construct the interaction among households.

Our future research work will focus on:

1. Researching the quantitative method and model, especially the scenario analysis model which may reflect the interaction among different household types.
2. Seeking for more gentle risk adverse principle and increasing the rotate crops condition between fields is one aim of our future research.
3. Exploring the methods in which household decision-making over a longer period can be researched, which also allows us to find the bridge between lots of LUCC data and the limited household making-decision.
